# Scanning Electrochemical Microscopy for Chemical Imaging and Understanding Redox Activities of Battery Materials

**DOI:** 10.1021/cbmi.3c00014

**Published:** 2023-03-23

**Authors:** Lyndi E. Strange, Xiao Li, Eric Wornyo, Md Ashaduzzaman, Shanlin Pan

**Affiliations:** † Pacific Northwest National Laboratory, Energy and Environment Directorate, 902 Battelle Blvd., Richland, Washington 99352, United States of America; ‡ The University of Alabama, Department of Chemistry and Biochemistry, 250 Hackberry Lane, Tuscaloosa, Alabama 99354, United States of America

**Keywords:** SECM, SEI, battery, scanning probe imaging, LIB, *in situ* imaging, electrochemical characterization, energy storage development, redox-flow battery

## Abstract

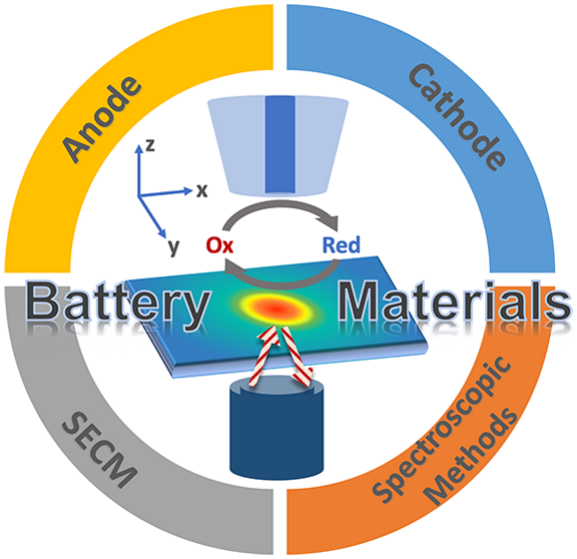

Improving the charge storage capacity and lifetime and charging/discharging efficiency of battery systems is essential for large-scale applications such as long-term grid storage and long-range automobiles. While there have been substantial improvements over the past decades, further fundamental research would help provide insights into improving the cost effectiveness of such systems. For example, it is critical to understand the redox activities of cathode and anode electrode materials and stability and the formation mechanism and roles of the solid–electrolyte interface (SEI) that forms at the electrode surface upon an external potential bias. The SEI plays a critical role in preventing electrolyte decay while still allowing charges to flow through the system while serving as a charge transfer barrier. While surface analytical techniques such as X-ray photoelectron (XPS), X-ray diffraction (XRD), time-of-flight secondary ion mass spectrometry (ToF-SIMS), and atomic force microscopy (AFM) provide invaluable information on anode chemical composition, crystalline structure, and morphology, they are often performed *ex situ*, which can induce changes to the SEI layer after it is removed from the electrolyte. While there have been efforts to combine these techniques using pseudo-*in situ* approaches via vacuum-compatible devices and inert atmosphere chambers connected to glove boxes, there is still a need for true *in situ* techniques to obtain results with improved accuracy and precision. Scanning electrochemical microscopy (SECM) is an *in situ* scanning probe technique that can be combined with optical spectroscopy techniques such as Raman and photoluminescence spectroscopy methods to gain insights into the electronic changes of a material as a function of applied bias. This Review will highlight the potential of SECM and recent reports on combining spectroscopic measurements with SECM to gain insights into the SEI layer formation and redox activities of other battery electrode materials. These insights provide invaluable information for improving the performance of charge storage devices.

## 1 Introduction

**Figure 1:**
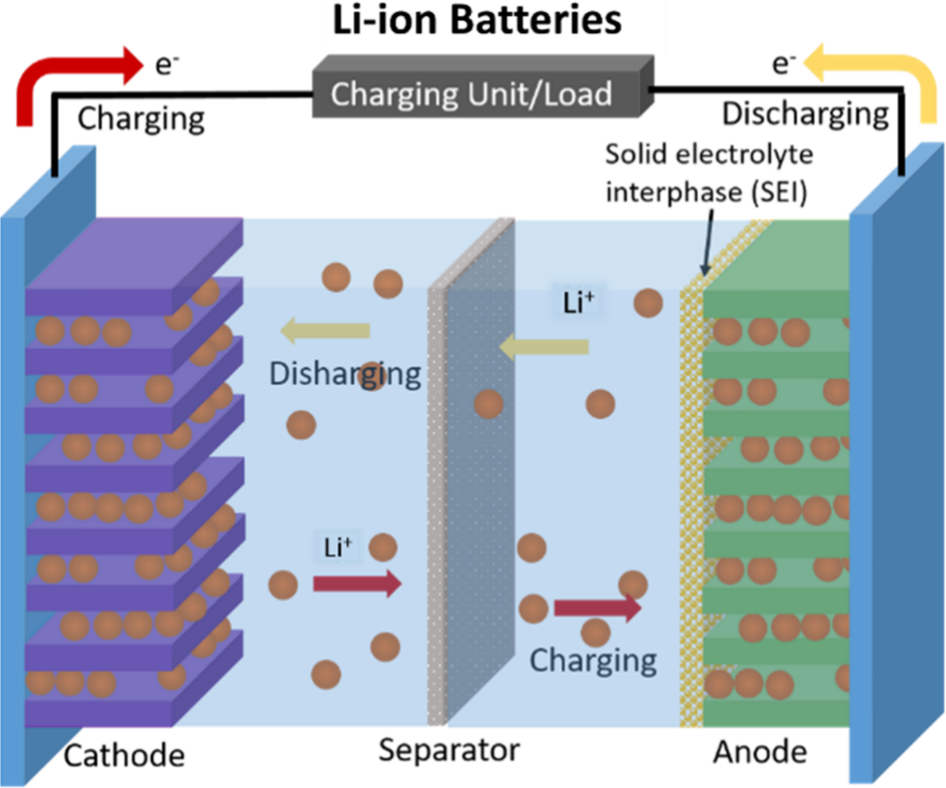
Schematic of a LIB including a cathode, anode, and separator.

Finding more efficient methods of electrical energy storage is essential for modern society. Over the past two decades, the efficiency of lithium ion batteries (LIBs) has increased 3 times since the early 1990s,^1,2^ which has resulted in extensive applications in portable electronics and grid storage. Although the manufacturing cost of Li batteries has been reduced, further cost decrease is needed for affordable large-scale production, especially for long-range vehicle driving.^3^ Generally, LIBs consist of a cathode, anode, and separator. The separator prevents electrical contact between the cathode and anode and serves to facilitate ion transfer through the liquid electrolyte without direct electrical contact. Currently, the chemistry for Li batteries is developing with lithium nickel manganese cobalt (Li-NMC) used as the cathode material and graphite for the anode. The LIB works by the transport of Li ions across the separator upon an external bias to equilibrate the charges in the system. Specifically, the Li ions migrate from the negative side (graphite) to the positive side (NMC) through the liquid phase and separator. Since Li metal is scarce and highly reactive, other types of batteries are among the growing battery research areas with the potential to serve as alternatives to LIBs for large-scale energy storage. For instance, sodium,^4^ potassium,^5^ and aluminum^6^ ion batteries have received much attention due to their comparable capacity.^7^ The mechanism of these new types of batteries mimics that of a LIB (Figure 1) except with Na^+^, K^+^, and Al^3+^ ions to replace Li^+^. Furthermore, these metal ions offer a lower standard reduction potential than Li^+^, which can be further lowered depending on the solvent used for the electrolyte. However, in these alternative metal ion batteries, the larger ionic radius of the metal ions results in lower ionic diffusion compared to Li^+^, which can lead to volume variations causing electrode pulverization and poor cycling performance. Therefore, understanding the charging and discharging mechanism in battery systems is essential to device development.

During the charging and discharging of all battery systems, a solid–electrolyte interface (SEI) layer forms at the liquid–solid interface of the anode, which greatly affects performance and is considered one of the least understood components of battery systems due to the lack of *in situ* experimental methods.^8^ Furthermore, understanding and modifying the SEI layer of battery systems is considered key to performance improvement since it is where the metal cation gets stored in the electrode via intercalation, alloying, or electrode metal. The SEI layer was first discovered by Dey^9^ et al. by examining lithium-soaked metal in nonaqueous electrolyte and further investigated by Peled^10^ who coined the term SEI by examining the passivation layer that formed on the negative electrode at the solid–electrolyte interface. The SEI layer allows Li^+^ or K^+^ transport and blocks electrons to prevent electrolyte decomposition and prolong cycling stability. Understanding the mechanism of formation as well as chemical and morphological changes on the surface is imperative to the lifetime improvement of battery systems.

Surface analytical techniques such as X-ray photoelectron (XPS), secondary ion mass spectrometry (SIMS), and X-ray diffraction (XRD) are invaluable tools to investigate the before and after changes of an anode surface before and after electrochemical bias. Oxidation state changes as well as elemental quantification can be achieved with XPS with extremely high surface sensitivity but sometimes is prone to data analysis errors.^11,12^ Time-of-flight secondary ion mass spectrometry (ToF-SIMS) can provide 2D images of Li distribution of an anode surface, which can provide insights to transport mechanisms during potential bias.^13^ Usually, ToF-SIMS and XPS are performed after electrochemical cycling, which may damage the SEI layer during rinsing (even in an inert atmosphere). Therefore, developing methods capable of gaining insight into the electronic behavior (i.e., charge transfer mechanism) at the electrode–electrolyte interface under potential bias is essential to understanding the charging/discharging mechanism from both an electrochemical standpoint and a surface mechanistic point-of-view. *In situ* analytical methods provide several advantages *in operando* or *ex situ* since they allow probing of the environment in the actual electrolyte under applied potential bias. One such method is scanning electrochemical microscopy (SECM), which is a scanning probe technique that leverages an ultramicroelectrode (UME) that receives electrochemical feedback from the surface (discussed in more detail below) and has been used in several applications to investigate the charging/discharging mechanism of battery anodes.^14^ Other analytical techniques including Raman spectroscopy have been proven to be an invaluable tool to investigate the solvation of Li ions at the SEI layer,^15^ structural ordering around the SEI layer,^16^ and Li metal intercalation mechanism.^17^ However, there have been few reports that have shown SECM in conjunction with a spectroscopic method such as Raman spectroscopy to examine the SEI layer.

## 2 Operation Principles and Instrument Capability of SECM

**Figure 2:**
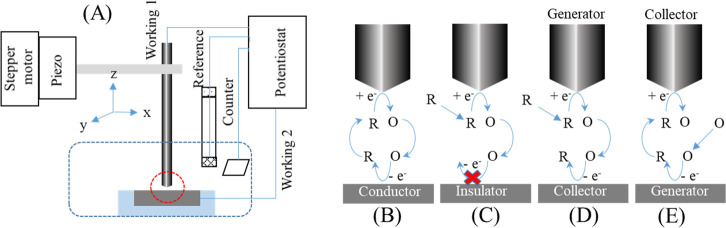
(A) Schematic of a typical SECM with the *x*–*y*–*z* positioner connected to a stepper motor and a piezo positioner that is controlled by the PC along with a 4-electrode system connected to a potentiostat. Schematic of SECM and its operation in various modes including (B) positive feedback, (C) negative feedback, (D) TG/SC, and (E) SG/TC mode.

In SECM imaging (Figure 2), spatially resolved information on a surface is obtained by monitoring the tip current as a function of the tip position.^18,19^ The tip electrode is moved stepwise in the *z*-direction onto the surface of a substrate until it is very close to the surface (enough to receive current feedback). The substrate surface is scanned in the *x*–*y* direction while holding the tip electrode (either micrometer or nanometer in diameter) at a constant height (*z*-direction).^20^ Electrochemical information about each surface location is obtained due to the current measured at the tip electrode, which is displayed as colored patterns with each color corresponding to a respective current value. SECM feedback mode (FB-SECM, Figure 2B,C) is the most used SECM imaging mode for the characterization of a surface. In this mode, the oxidation and reduction of a redox mediator added to an electrolyte solution shows either an increase or a decrease in current at the tip electrode as the tip is polarized at a potential where the mediator reaches a limiting diffusion current.^20,21^ The increase in current is termed positive feedback, which is due to the tip electrode approaching a conductive substrate whereas the decrease in current is termed negative feedback, which is due to the tip electrode approaching an insulative substrate.^21^ Another mode used in SECM imaging is the substrate generation/tip collection (SG/TC) mode^20^ where electrochemically active molecules generated at the surface are detected by the tip electrode as shown in Figure 2E. This results in an image map showing current intensity as a function of electrode reaction distributions. Kang et al. used the FB-SECM mode to obtain an SECM image that reflects the surface topography, reactivity, and the formation of the solid electrolyte interface (SEI) on the graphite anode electrode of LIBs.^22^ The feedback images were recorded by taking an area scan of 120 μm × 120 μm. They were able to observe a spontaneous reaction on the surface of the electrode with the formation and evolution of an SEI during and after cycling. Li and co-workers also investigated the evolution of SEI in a concentrated aqueous electrolyte on a C-TiO_2_ anode electrode by obtaining SECM images of a 144 μm × 144 μm area using the feedback mode.^19^ They observed SEI components that were randomly distributed over the surface of the electrode.

### 2.1 Substrate Generation/Tip Collection (SG/TC)

The SG/TC mode (like the feedback mode) is among the earliest mode of SECM to be introduced in 1989.^23^ This mode allows the generation of an electroactive species at the substrate surface, which is collected or detected at a biased tip electrode. Information regarding the chemical flux at the substrate surface is obtained by measuring currents at the substrate and the tip electrode.^24^ The SG/TC mode has seen some applications in the study of LIBs. For example, Xu et al. used this mode for the *in situ* studies of Li ion transportation at the interface of the LiCoO_2_ electrode during the charging process.^25^ In this study, they chose a higher conductive electrolyte, an oxidation potential of LiCoO_2_ at the SG condition, a reduction potential for Li^+^ ions dissociating from LiCoO_2_ at the TC condition, and a distance between the tip electrode and substrate. They observed some variations in the concentration of Li^+^ ions at the tip electrode because of the breakdown of solvated Li^+^ ions originating from the LiCoO_2_ electrode. In another SG/TC SECM mode application, Snook et al. were able to detect solubilized Co^2+^ generated from the LiCoO_2_ electrode at a high positive potential.^26^ The tip electrode biased at a potential and maintained at a constant position was used to collect Co^2+^ ions.

### 2.2 Surface/Substrate Interrogation

**Figure 3:**
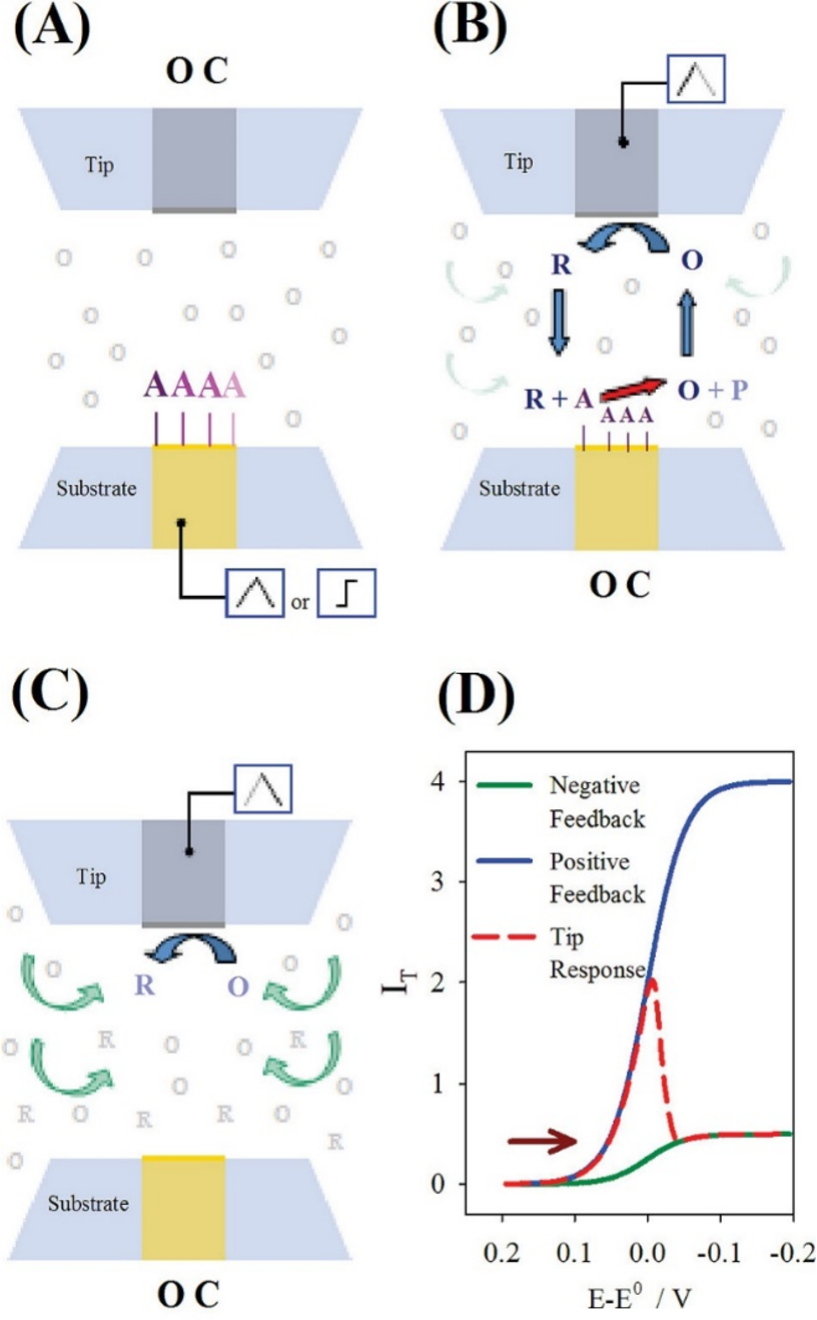
Schematics of the operation principle of the surface interrogation method. (A) A reactive species is electrochemically adsorbed at the substrate upon a potential scan while the tip is at an open circuit. (B) The substrate is taken to open circuit, and the tip generates the titrant to react at the surface of the substrate to support positive feedback at the same tip. (C) Upon consumption of the adsorbate at the substrate, the tip experiences negative feedback. (D) Expected current response at the tip following the events depicted in panels A–C for an arbitrary electrode setup. Figure reproduced from ref (27). Copyright 2008 American Chemical Society.

In the surface or substrate interrogation SECM (SI-SECM) mode, a transient positive feedback current is recorded at the tip electrode due to electron transfer between the redox mediator and species bound onto an electrode surface.^27^ To demonstrate the capabilities of SI-SECM, Bard et al.^27^ interrogated platinum and gold electrode surfaces in order to measure species that were chemisorbed. An oxidation reaction on the substrate occurs by applying a potential that leads to the formation of a species (species A) that is chemically bound onto the surface while keeping the tip electrode at an open circuit (Figure 3A). Under these conditions, a redox mediator already in the oxidized form (species O) does not participate in the reaction. The potential at the substrate and tip electrode is then reversed by keeping the substrate at an open circuit while applying a scanned potential to the tip electrode. This leads to the reduction of species O to its reduced form (species R) known as a titrant. The titrant diffuses across the tip–substrate distance (approximately 1 μm) and reacts with species A (Figure 3B). This results in the consumption of species A while species O is regenerated. A transient positive feedback loop at the tip electrode is produced as the reaction between species R and species A continues until species A is consumed completely (Figure 3C,D). Negative feedback is recorded as species A is consumed, and the rate of reaction of species O at the tip is limited by the diffusion in the gap between the tip and substrate surface.^27^ The contrast between the positive and negative feedback provides a sensing mechanism that will allow the neutralized charge at the substrate to be measured. In addition, with regard to the potential of the substrate and under open circuit conditions, the quantification of the bound species on the substrate surface can be achieved.^27,28^ This mechanism can be useful as a characterization method and for evaluating SEI formation on the surface of anode electrodes due to its sensitivity to surface charging and discharging.

### 2.3 *In Situ* Combination Using IR and Raman with SECM

SECM as a standalone technique can provide information about the physical and chemical changes occurring on the electrode surface. Thus, the electrochemical reactivity on the electrode surface that is observed correlates directly to the SEI electronic characteristics.^29^ However, combining SECM with other *in situ* techniques will allow the testing environment in which the battery material is analyzed to be maintained. This will provide an invaluable means of correlating changes in the properties of the battery material or SEI in real-time.^13,30^ Coupling *in situ* spectroscopic techniques such as Raman and IR can provide molecular information about the interfacial reaction that correlates the interfacial local structure to the electrochemical reactivity. Schuhmann et al. investigated the local surface modification of a nanostructured gold electrode surface by coupling SECM to Surface Enhanced Raman Scattering (SERS).^30,31^ With this technique, the SECM tip reduced para-nitrothiophenol to para-aminothiophenol on the nanostructured gold electrode surface. The results obtained showed changes in the SERS spectra from a self-assembled monolayer (SAM) and a change in the measured feedback SECM current, which is due to the redox activity of para-nitrothiophenol. The information obtained from the SERS measurement will enable the interfacial electrochemical reactions to be probed while providing interfacial molecular identities.

**Figure 4:**
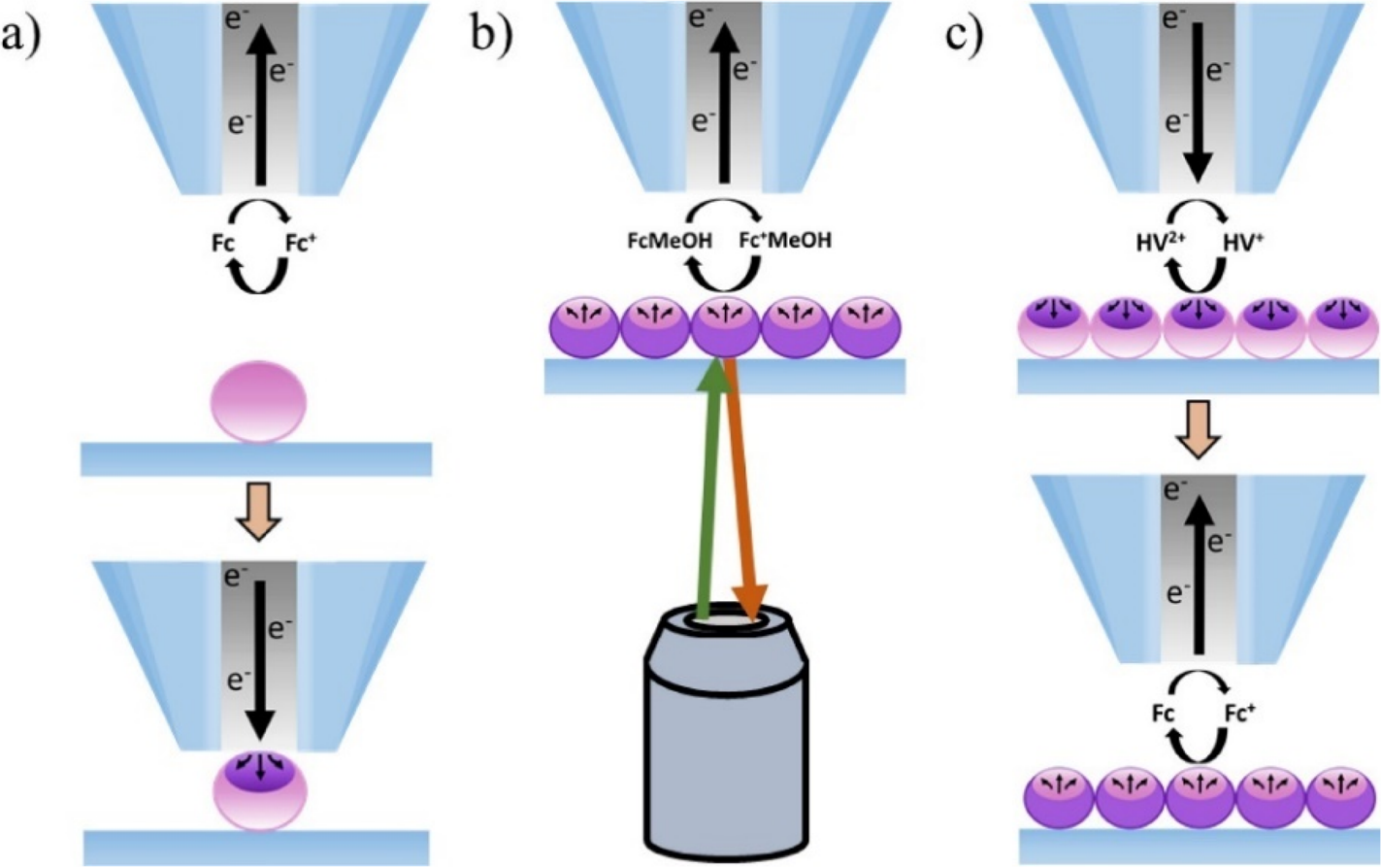
Schematic of the experimental setup used by Gossage et al.^33^ (a) Shows the tip approaching a single particle; (b) shows the incorporation of the Raman spectroscopic setup; (c) shows oxidation/reduction measurements in the surface interrogation (SI) mode of SECM. Adapted from ref (33). Copyright 2017 American Chemical Society.

Previous reports pertaining to Raman coupled with SECM include Schorr et al., who examined the interfacial activity of graphene via SECM with Raman spectroscopy to examine localized charge transfer at the solid–electrolyte interface,^32^ and Gossage et al., who used the same technique to understand charge transport through redox-active colloids using the experimental set up shown in Figure 4.^33^ Additionally, Thangavel et al. used *in situ* atomic force microscopy (AFM) in conjunction with SECM to investigate topographical changes of lithium/sulfur battery anodes during oxidation.^34^ While these reports provide invaluable insight into the battery charging/discharging mechanism, there is room to grow to combine SECM with surface-sensitive spectroscopic techniques to elucidate the charge transfer and formation mechanism of the SEI layer on battery anodes.

In an IR/SECM combination technique, Kranz et al. studied an *in situ* electrochemically induced process by using a single bounce attenuated total reflection (ATR) ZnSe crystal as SECM substrate.^35^ They demonstrated the capabilities of this technique by the spectroscopic monitoring of microstructured electropolymerization of the 2,5-di(2-thienyl)-pyrrole (SNS) layer induced by SECM feedback mode on the surface of a ZnSe crystal. The polymerization reaction was monitored electrochemically by a Ru(bpy)_3_^2+^ mediated feedback SECM current and by monitoring the changes in absorption intensity of SNS IR bands. The information obtained from the IR spectra could be synchronized with the electrochemical data to provide information about the polymerization mechanism and surface modification. Therefore, spectroscopic techniques such as IR and Raman in combination with SECM have the possibility of providing molecular information that is necessary to characterize interfaces during electrochemical measurements.

## 3 Applications of SECM to Study SEI Layer Formation on Anode

**Figure 5:**
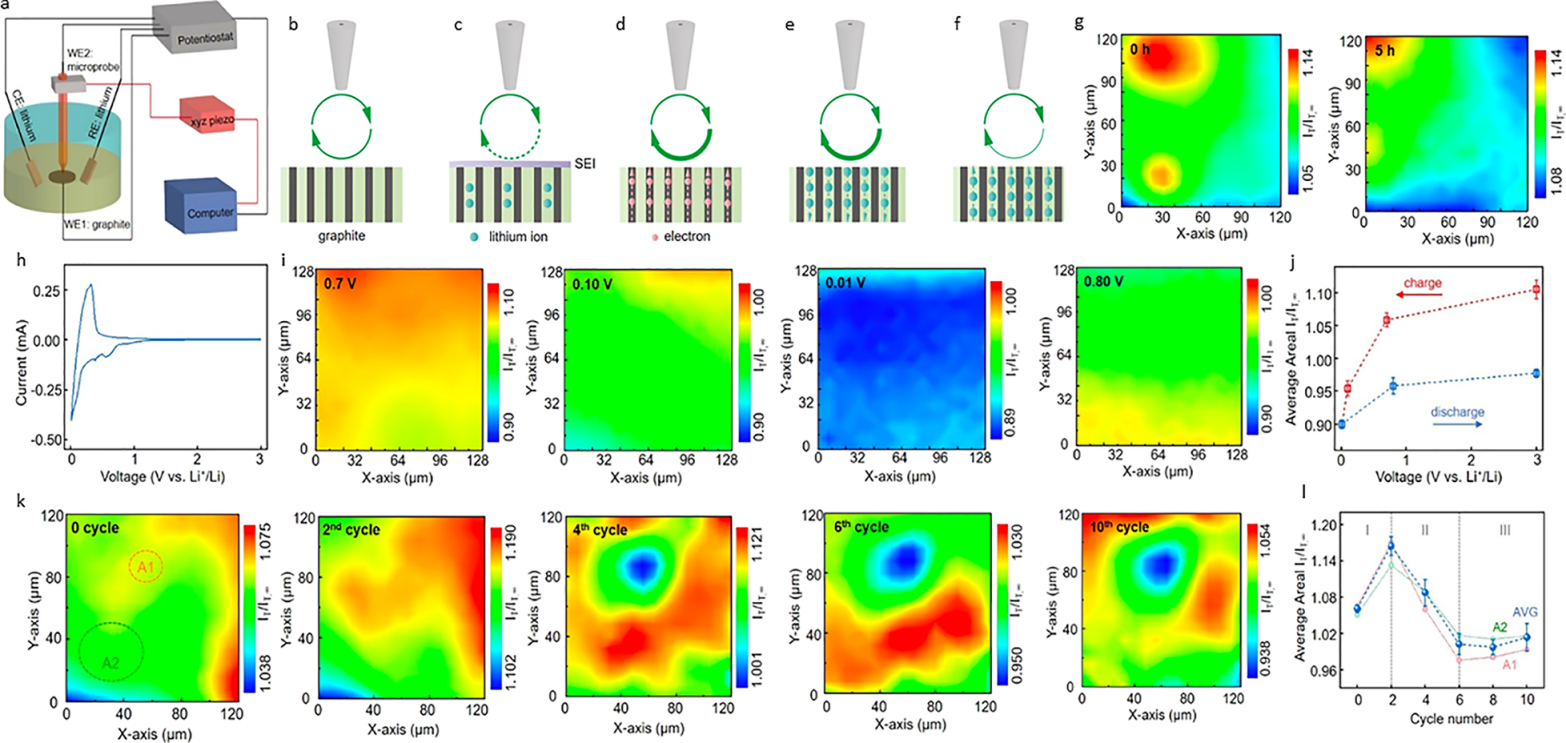
Characterization of SEI and interfacial electrochemical properties of a graphite anode for LIB by SECM. (a) Schematic of SECM setup followed by schematics of SECM used in both positive and negative modes. (b) Positive feedback mode of pristine graphite whereas negative feedback mode is shown for (c) pristine graphite, (d) SEI formation, (e) substrate driving force effect, I lithiation, and (f) delithiation. (g) Area scans with a tip bias of 3.5 V (vs Li/Li^+^) before and after immersion in 1 M LiPF_6_/EC/DEC/EMC for 5 h. (h) CV of graphite electrode at a scan rate of 1 mV/s. (i) Area scans recorded at graphite potential from 0.7 to 0.01 to 0.8 V (vs Li/Li^+^). (j) Change of average area relative current *I*_T_/*I*_T,∞_ with electrode potential. (k) Area scans of pristine graphite and after 2nd to 10th cycles. (l) Average relative current *I*_T_/*I*_T,∞_ with the cycle number. Adapted from ref (22). Copyright 2020 American Chemical Society.

A robust SEI layer formed at the anode surface can prevent further loss of active material by electrical insulation and keep the cycling integrity of the layered anode structures.^2,36−38^ Graphite is one of the most commonly used battery anode materials, and its dynamic surface aging behaviors in the presence of SEI have been studied by using SECM.^39^ In this case, the SECM probe works in a nondestructive feedback mode, which generates electrochemical signals with respect to the interfacial electrochemical properties such as surface conductivity, potential driving force, and interfacial lithium migration effect.^39−41^ Both Bülter et al. and Zeng et al. (Figure 5) have applied SECM imaging to study the spatiotemporal formation and evolution of SEI on the uncharged and cycled graphite anodes for LIB.^39,41^ Spontaneous dynamic processes such as volume change, dissolution, and gas evolution under no external bias were revealed in reactivity variations at some particular surface spots.^39,41^ Insulating SEI was formed in the cathodic sweeps of the first few cycles followed by an areal decomposition during the delithiation process.^39^ Bülter et al. have reported the use of SECM for investigating SEI evolution upon certain rinsing protocols of a lithiated graphite anode for the *ex situ* characterization.^40^ SEI passivity decreased accompanied by a reduced open circuit potential (OCP) due to the localized dissolution of specific components in SEI during the rinsing step.^40^ The long-term and short-term temporal variations of SEI passivity were studied. With respect to the bulk graphite, few-layer graphene (FLG) that shows a layer-number-dependent staging Li^+^ intercalation mechanism has been imaged by Hui et al. using SECM.^42^ A Hg-capped Li^+^ sensitive ionic probe was employed for illustrating the preferred Li^+^ intercalation on the exposed edge plane of graphene.^42^ Transition metal oxides are also important candidate anode materials, where SECM has been a powerful tool for elucidating their distinct battery charge mechanisms.^43,44^ For instance, a protecting and passivating SEI layer was only found on TiO_2_ cycled at a relatively lower-voltage window (3.0–1.0 V vs Li/Li^+^, 1 M Li^+^ in EC: DEC) in aid of SECM and XPS, whereas the formation of an SEI-like layer under a higher-voltage cycling window (3.0–2.0 V vs Li/Li^+^) and during 6-week storage under nonmoisture contact in the electrolyte had no impact on the electrode surface reactivity.^44^ Liu et al. further reported the random distribution of SEI (composed of inorganic LiF, Li_2_CO_3_, and Li_2_O by XPS) over TiO_2_ anode in concentrated aqueous electrolytes by using feedback- and alternating current (ac)-SECM.^43^ However, it has brought up a concern that the variations in feedback kinetics contributed by surface reactivity and local topography cannot be thoroughly deconvoluted by the conventional feedback SECM imaging in a constant-height mode. Although the topographic effect could be ruled out by calibrating with the pristine state electrode surface,^43^ a more straightforward constant-distance imaging method of SECM is highly in demand in case there is simultaneous topography variation along with the change of surface reactivity. For example, a large volume change is usually involved during the conversion reaction of the transition metal chalcogenides battery anode during charging cycles.^45−47^ Takahashi et al. have recently utilized scanning electrochemical cell microscopy (SECCM), a variation of SECM using a nanopipette as the probe, to quantify the facet-dependent reactivity and diffusion coefficient of a Li_4_Ti_5_O_12_ thin-film anode surface.^48^ Simultaneous topography and current images were provided by applying a constant voltage, where a metastable state of LiFePO_4_ could be identified.

Up to now, there are still limited applications of SECM for characterizing battery anodes due to the complexity of experimental setup and operation. The battery electrode surface has to be open for accommodating the SECM probe, which highly requires inert ambient and proper electrode preparation and storage protocols. It is also worth noting that the redox environment generating detectable feedback signals in highly reactive lithium battery systems needs to be carefully optimized. 2,5-Di-*tert*-butyl-1,4-dimethoxybenzene (DBDMB) has been raised as a more appropriate redox mediator compared to the commonly used ferrocene (Fc) in SECM studies for battery anodes.^40,41^ The electron transfer of Fc^+^ to Fc can be more strongly inhibited by the SEI layer resulting in a quick loss of tip current once the electrode surface is covered. Fc derivatives are also reactive with Li metal. DBDMB gives a much more gradually decreased electron transfer kinetics upon SEI formation and benefits long-term spatiotemporal observation. Furthermore, there are difficulties in excluding the interference from surface species’ adsorption/desorption at the SECM probe and assigning the chemical nature of interfacial reactions by simply relying on the electrochemical signals.^39^*In situ* supplementary chemical evidence is thus needed for correlating the surface reactivity to the structural changes of a battery electrode with the aid of spectroscopic tools.

## 4 Applications of SECM to Image Battery Cathodes

**Figure 6:**
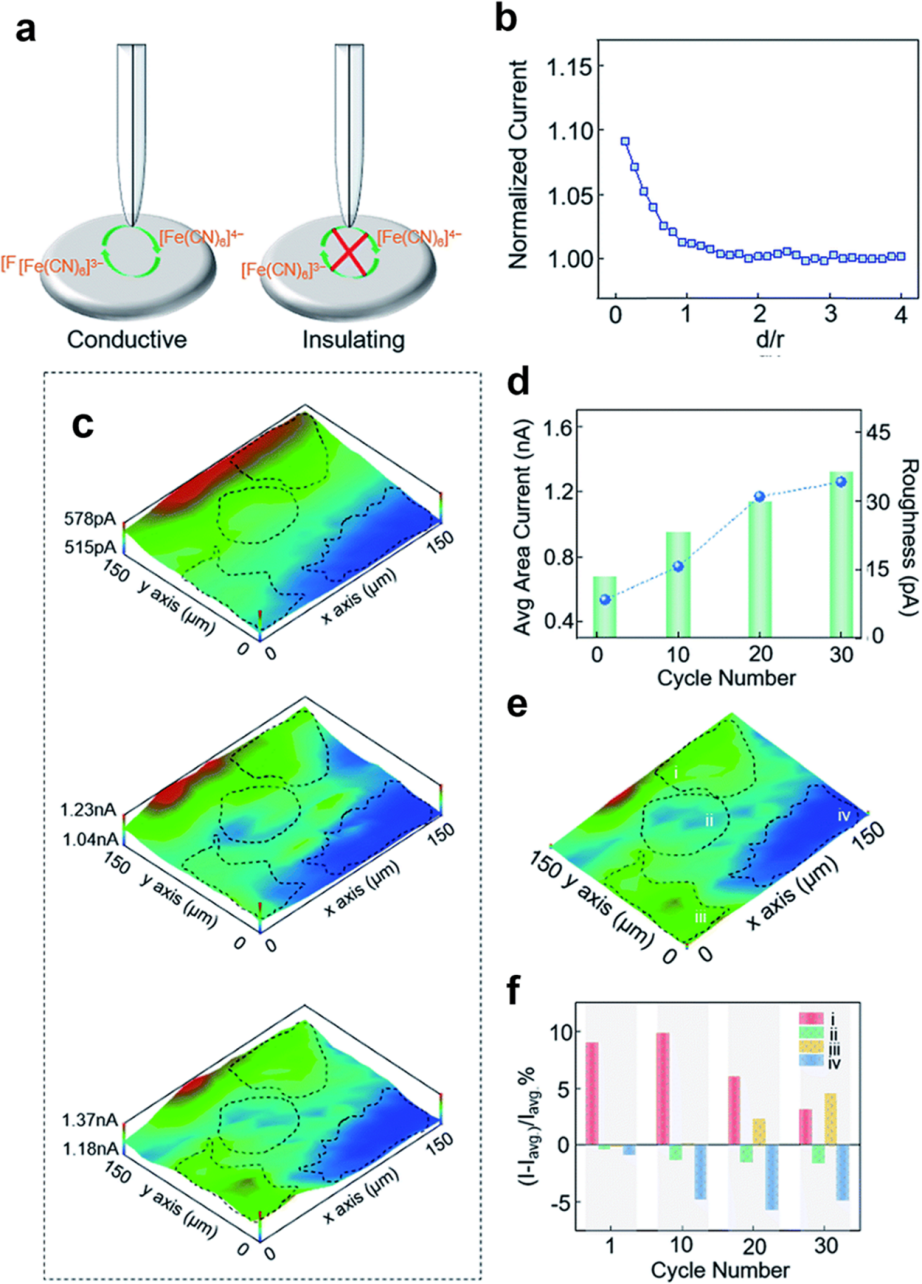
Schematic presentation of positive and negative feedback mode of SECM (a), SECM approach curve at LiMn_2_O_4_ (b), SECM area scan with feedback mode at different cycles (c), average area scan feedback current density and feedback current roughness change with cycles (d), four different conductive areas at the substrate surface (e), and difference of feedback currents with the average area current change with cycles in four regions (f). Adapted with permission from ref (54). Copyright 2019 Royal Society of Chemistry.

Like the anode, the cathode is another important part of LIB to get batteries with high energy and power density as well as longevity. The choice of a perfect cathode material with a particular surface chemistry depends on various factors including cell voltage, capacity, energy and power density, and operation conditions. Cathode materials of LIBs are mainly lithium-containing transition metal oxides (i.e., Lithium Manganese Oxide (LMO), Lithium Cobalt Oxide (LCO), Lithium Nickel Oxide (LNO), Lithium Iron Phosphate (LIP)).^49^ These materials have their advantages and disadvantages regarding the electrochemical environment in the cell. Structural reconstruction, morphological changes, and cathode surface electrochemistry greatly affect the battery performance.^50^ The main reasons for deteriorating battery performance with the charging/discharging cycle are attributed to capacity loss due to surface deoxygenation and transition metal dissolution from the cathode’s surface along with electrolyte decomposition.^51^ This can induce different structural and chemical properties to the cathode surface with time. Although the LIB anode’s surface chemistry has been extensively investigated, a comprehensive understanding of the cathode’s surface chemistry has yet to be achieved. Various *ex situ* techniques (i.e., SEM, TEM, AFM, XRD, and different spectroscopy methods) are extensively employed for surface characterization and surface chemical identification, which are limited by real-time surface chemistry information in an actual electrochemical environment. SECM can be a useful technique for the *in situ* understanding of LIB cathode material’s surface chemistry. SECM can detect solubilized species of low detection limits at the surface with low concentration. SECM cathode material studies mainly focused on transition metal dissolution from cathode materials during the charging cycle. Snook et al. employed SECM to investigate the direct solubilization of species leaving the LiCoO_2_ cathode materials of LIB in ionic liquid (i.e., 1-butyle-1-methylpyrrolidium bis(trifluoromethanesulfonyl)imide).^26^ A substrate generation/tip collection mode of SECM was employed in their study for surface chemical probing where a Pt microelectrode was placed near the cathode surface to detect the Co^2+^ ions leaving the cathode electrode. Solubilized Co^2+^ and oxygen were observed during the cell charging/overcharging and deep charging steps. Xu et al. employed the SG/TC mode of SECM to investigate the transportation of Li^+^ ions at the interface of a charging LiCoO_2_ and observed nonuniform electrolyte distribution over the electrode.^25^ Huang et al. used the SG/TC mode of SECM to detect leaving Mn ions from the LiMnO_4_ and reported significant evolution of Mn^2+^ ions from LMO with no Mn^3+^ ion detection.^52^ The same group fabricated a thin film LMO cathode and investigated Mn dissolution from LMO in different electrolyte systems (i.e., ClO_4_^–^, PF_6_^–^, and (CF_3_SO_2_)_2_N).^53^ A significant effect of electrolytes on Mn dissolution and the electrochemical behavior of the generated Mn complex was observed in the study. Liu et al. investigated the cathode–electrolyte interface with SECM (Figure 6) and observed a discontinuous cathode–electrolyte interface (CEI) with conductive properties at the LiMn_2_O_4_ cathode due to the result of salt decomposition using SECM feedback mode.^54^ Lattice oxygen loss during cathode charging significantly limits the charge storage capacity of LIB and subsequent surface reconstruction phenomena. Mishra et al. observed two-stage oxygen evolution behavior when commercial LIB cathode materials, transition metal oxide cathodes (TMOC), were studied using SECM. Additionally, a heterogeneity of oxygen evolution from different cathode locations was observed in the study with SECM mapping, which is consistent with the morphological heterogeneity of TMOC.^14^ Although SECM has shown great potential for the *in situ* surface chemical investigation of LIB cathode materials, most studies have used SECM microelectrodes for chemical investigation and surface mapping. Thus, more work needs to be done to get a comprehensive insight into the cathode materials reconstruction and transition metal dissolution using SECM tips within the nanometer dimension to achieve a high special resolution SECM mapping of the surface.

## 5 Other Structure/Electron Transfer Studies with SECM

**Figure 7:**
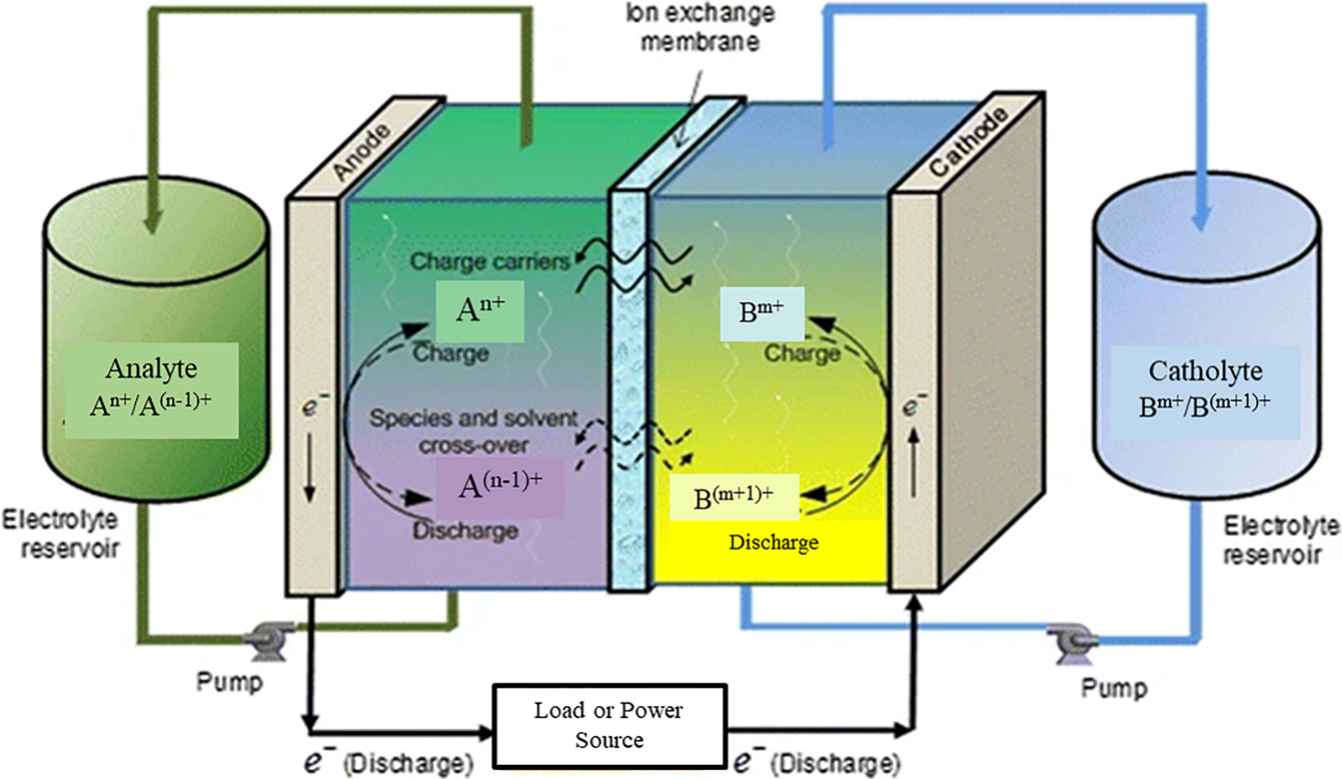
Schematic diagram of the redox flow battery system with electron transport through the circuit, ion transport through the electrolyte and membrane, active species crossover, and electrolyte flow. Adapted with permission from ref (55). Copyright 2011 Springer.

Redox-active polymers have been applied to both static and flow battery systems. Redox flow batteries (RFBs) are another class of battery systems that uses a continuous flow of electrolyte through the system (Figure 7). In a typical RFB system, there are two separate electrolyte tanks: one for the cathode (catholyte) and the second for the anode (anolyte). In the discharging mode of the RFB, the anolyte flows through a porous electrode where electrons are generated through an external circuit. The charge-carrying species are transported to an ion exchange membrane (IEM), which serves as a separator for the catholyte and anolyte solutions. The simplified electron transfer reactions for both the anode and cathode can be written as follows, respectively:^55^
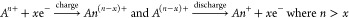
1
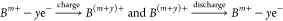
2

RFBs have several advantages over static systems for large-scale grid storage applications such as the ability to withstand a large number of charging/discharging cycles, high efficiency, the ability to respond quickly to changes in the system load, and reasonable production/maintenance costs.^55^ However, the major areas for development for RFB systems include charge transfer kinetics related to the electrolyte and crossover at the IEM, which can result in significant energy loss. Recently, nonaqueous redox flow batteries have shown more promising results than aqueous systems owing to larger voltage windows that can be utilized, a greater variety of redox molecules, and increased reaction potentials that can lead to energy-dense systems.^56,57^

SECM has also been utilized to understand the charge transfer mechanism of organic redox molecules for redox flow battery systems especially since there is a strong correlation between battery lifetime and the uptake of ions into the polymer film in an irreversible electron transfer reaction.^58^ Therefore, understanding the electron transport mechanism through the polymer is of utmost importance to improve the reversibility of the cation–electron transfer reaction. Traditional methods such as bulk analysis cyclic voltammetry allow insight into the overall reversibility of the redox reaction whereas SECM provides the capability to probe the ion transport throughout the film, which is essential for understanding the electron transfer mechanism. The first report of quantifying ion selective permeation via SECM was performed by Williams et al. where the molecular transport rates for patterned and porous substrates were calculated with substrate generation/tip collection mode as well as imaging to determine redox mediator permeation, which showed the versatility of SECM to quantify electron transfer reactions through various media.^59^

**Figure 8:**
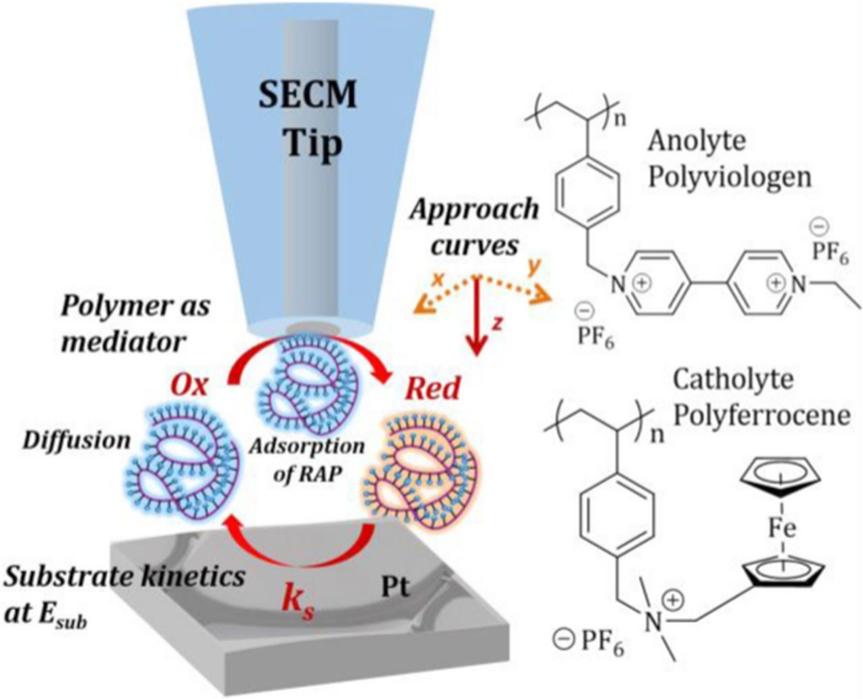
Schematic showing redox active polymers used as mediators for SECM to measure electron transfer kinetics. Adapted with permission from ref (60). Copyright 2016 Journal of the Electrochemical Society.

There have been several subsequent studies that have examined polymers in the context of RFB systems. Burgess et al. combined a SECM approach curve technique (Figure 8) with a rotating disk electrode (RDE) to obtain quantitative electron transfer mechanistic information about viologen-based redox active polymers.^60^ The results obtained in this study suggested solution-phase adsorption/desorption played a dominating role when compared to charge transfer alone. Additionally, Gossage et al. used a combined Raman/SECM approach to elucidate the charge transfer process through a redox active molecule containing viologen. The SI-SECM technique allowed the decoupling of charge diffusion to the probe and redox probe concentration whereas Raman spectroscopy allowed for the decoupling of diffusion and concentration as well as charge transfer tracking through the molecule by monitoring the intensity of the characteristic Raman peaks.^33^ This study showed that the combination of SECM coupled with a spectroscopic method such as Raman can elucidate and decouple various surface electron transfer processes occurring at the surface, which will aid in the development of charge transfer storage mechanisms in complex polymer systems.

## 6 Weaknesses and Strengths of SECM

As surveyed in this Review, SECM has demonstrated unique strengths in studying local redox events of major battery components including anode and cathode and electrolytes and membranes by illustrating their reactivities. The results of these investigations will provide insights into developing next-generation batteries with improved durability and charge storage capacity. Surface reactivities and the product of these reactions can be detected rapidly with the SECM probe. SECM can work as a complementary analytical tool to other electrochemistry methods to obtain an improved understanding of these surface activities. SECM imaging capability would provide spatial heterogeneities in local reactivities that are relevant to cell performance.

There are several major weaknesses of current SECM methods as listed below:

1. Most SECM configurations used for battery study are limited to a fixed imaging distance as shown in all examples, but the complexity of a cell’s components and structure would need analytical tools that can analyze them with constant height to their surfaces to obtain both topographic and electrochemical activity information, although there is still a lack of obtaining real 3D redox information on solid–liquid interfaces, especially those pertaining to the SEI layer of battery materials.
2. Stable substrate current with a minimum background current cannot be obtained directly via conventional micro- or nanoelectrode-based SECM, although the tip current can indirectly obtain high spatial resolution images of a battery substrate. One could obtain substrate current directly by creating a small sample surface area via patterning the sample surface to obtain individually addressable electrodes (e.g., via e-beam or photolithography), but the sample preparation process is slow and information on only limited regions can be obtained. Recent studies show that there is no limit to developing electrodes with various shapes and combinations such as duel or multiple channel probes for simultaneously collecting redox reaction products and sending redox intermediates to battery materials’ surfaces and interfaces to overcome the issues of conventional SECM methods. For example, alternative scanning electrochemical imaging methods such as SECCM^48,61^ would address the limitation of conventional microelectrodes by utilizing a pipet with a macroscopic reference electrode inside and liquid electrolyte meniscus at its orifice in contact with a battery surface. Redox activities of a battery material can be obtained with an improved spatial resolution and minimum background current from the substrate.
3. Although IR and Raman spectroscopy methods are applied to understand the surface and interfaces of battery materials in combination with SECM, there is a lack of local chemical information and their time evolutions, and SECM itself only provides current and potential information that is indirectly relevant to chemical reactions but does not provide direct chemical structural information. There is a need for Raman and IR spectroscopy techniques with an improved spatial resolution for probing local surface chemical characteristics. These optical methods can provide real-time chemical evolution of battery surfaces when applied to SECM *in situ*.
4. The complexity in battery geometry and types of cells such as solid and redox flow cells prove new opportunities and challenges for energy storage. SECM will continue to provide useful information for these batteries’ materials and their activities while the problem can be quite challenging and extreme such as high temperature and pressure. New SECM configurations and operation methods need to be developed to study batteries under these conditions.

## 7 Conclusion

SECM has become one of the most widely used electroanalytical techniques for studying electron transfer processes of energy materials. Many different analysis techniques can be utilized with the nanoprobe tip and surface to obtain a plethora of information. While surface analytical techniques such as XPS, SIMS, XRD, etc. provide invaluable information about the morphology, surface composition and oxidation state, and crystallographic orientation, these techniques are often performed *ex situ* and *operando*, which can result in some surface changes due to changing the surface atmosphere of the electrode. However, some methods have been developed to obtain as close to *in situ* data as possible using ultrahigh vacuum systems but have a focus on surface characterization rather than *in situ* electron transfer reactions.^62,63^ In order to gain a full understanding of the electrode surface properties, electroanalytical techniques are needed to elucidate the electron transfer properties. SECM has become a versatile technique used for a number of different systems including corrosion,^64,65^ electrocatalysis,^66^ and photoelectrocatalyis^67^ among many others. In order to gain a thorough understanding of the surface electron transfer reactions, SECM has been coupled with techniques such as Raman spectroscopy for more complex systems such as polymer-based RFBs (discussed in Section 5). While there have been a lot of advances in coupling SECM with Raman spectroscopy, there still is room to advance to other systems such as understanding the SEI layer that forms on battery anodes, which could aid in the development of these systems. While the SEI layer is extremely system dependent, the optical system utilized for spectroelectrochemical studies is usually versatile enough to adjust. This Review has pointed out the major studies involving battery-related materials and more specifically the SEI layer that forms during electrochemical cycling since it greatly affects the longevity and stability of all battery-related systems. Since the SEI layer forms at the interface of the solid electrode surface and the anode, *in situ* analytical techniques are useful to understand the chemical properties.

## Vocabulary

Scanning Electrochemical Microscopy (SECM): Scanning probe technique that utilizes an ultramicroelectrode probe (usually 10 μm) to understand the electronic properties of a surface through various techniques.
Substrate Generation/Tip Collection (SG/TC) Mode: A specific mode of SECM that cycles the potential of the substrate in a predetermined range to produce products (i.e., H_2_), which is then picked up at the ultramicroelectrode tip using the reverse electron transfer reaction (i.e., H_2_ produced at the substrate is oxidized at the tip). Substrate Collection/Tip Generation (SC/TG) can also be used.
Surface Interrogation (SI) Mode: A method similar to the SG/TC mode except focusing on adsorbed surface species of the electrode’s surface using electrochemical titration and spectroelectrochemical methods, for example.
Solid Electrolyte Interface Layer: When a potential is applied to a LIB, Li^+^ ions move across the membrane and can accumulate at the anode surface. Many electrolytes contain a variety of species that can precipitate out to form a solid layer on top of the anode surface, which affects the battery’s performance and long-term stability.
Ultramicroelectrode: The probe used in SECM that usually consists of a Pt (<10 μm) wire encapsulated in a glass capillary tube that is used to probe the electronic properties of a surface.
